# Linking UV-induced DNA damage with base pair sequences

**DOI:** 10.64898/2026.05.05.722932

**Published:** 2026-05-08

**Authors:** Luc Wieners, Martin E. Garcia

**Affiliations:** Institute of Physics, University of Kassel, 34132 Kassel, Germany

## Abstract

Ultraviolet (UV) radiation induces DNA damage associated with cancer and aging, yet the sequence dependence of UV absorption remains to be investigated. Here, we present a systematic study of the UV absorption spectra of DNA based on all-electron Hartree–Fock calculations. We analyze all possible sequences up to four base pairs, as well as longer randomized sequences and genomic nullomers – motifs which are missing in a given genome. We observe a pronounced sequence dependence: cytosine- and guanine-rich motifs exhibit significantly enhanced absorption, whereas adenine–thymine-rich sequences absorb up to four times less in the mid-UV range. Notably, the human genome is biased toward adenine–thymine-rich sequences, giving it an increased susceptibility to UV-induced damage. In addition, we introduce a computational framework enabling spectral calculations of large DNA and RNA fragments, opening the door to large-scale optical analyses.

The human genome contains around 3 billion base pairs which are the smallest units of information in DNA, each containing around 60 atoms. This information is continuously exposed to environments which can damage it, with estimates of around 10,000 instances of DNA damage occurring per cell each day [1]. Evolution has given rise to numerous DNA repair mechanisms that, in most cases, mitigate damage caused by environmental and endogeneous agents [2]. However, in situations where DNA repair fails, the sustained DNA damage can lead to aging and cancer.

Among the environmental causes of DNA damage, ionizing radiation plays an important role, especially in the form of ultraviolet radiation due to everyday exposure to the sun. UV radiation can directly cause damage by forming pyrimidine dimers [3] which break up the helical DNA structure or indirectly, by creating reactive oxygen species which may cause harm when reacting with DNA [4].

A quantitative understanding of how UV absorption influences the occurrence of DNA sequences in the genome is still lacking. In this Letter, we demonstrate using all-electron Hartree–Fock calculations that UV absorption in DNA is strongly sequence-dependent, with enhanced absorption in cytosine–guanine-rich motifs such as nullomers (DNA sequences which do not occur in the human genome) [5, 6].

Ultraviolet (UV) radiation is conventionally classified into UVA (315–400 nm), UVB (280–315 nm), and UVC (100–280 nm) bands according to wavelength. Solar UVC is almost completely absorbed by the atmosphere and does not reach the Earth’s surface in significant amounts. Consequently, terrestrial UV radiation consists primarily of UVA and UVB, with UVA dominating due to its weaker atmospheric attenuation. However, DNA exhibits negligible absorption at wavelengths above 320 nm; therefore, we focus on UVB radiation in this study.

To gain systematic insight into the UV absorption of DNA samples, we calculated the absorption spectrum of all possible DNA structures with up to four base pairs; this leads to four structures with one base pair, 16 with two, 64 with three and 256 structures with four base pairs. Additionally, we studied longer DNA samples with eleven base pairs. Here we investigated ten nullomers and ten of their synonyms - DNA sequences which are translated in the same way but have a different sequence of base pairs. As a reference group, we computed the spectra of ten random DNA sequences as well.

The DNA structures with one, two, three, and four base pairs contain approximately 60, 125, 190, and 250 atoms, respectively, while the DNA samples with eleven base pairs contain around 700 atoms each. However, fully quantum-mechanical absorption spectra calculations become computationally extremely demanding if a system size of 100 to 200 atoms is exceeded. In these cases, QM/MM methods [7] that combine molecular mechanics (MM) [8–11] with quantum mechanics (QM) [12, 13] in a core region are often used. Since we want to study the absorption of the complete DNA structure and not just a selected region, we instead treat the complete system with quantum mechanics and downscale the accuracy for computational feasibility.

Our used framework based on the real-time time-dependent Hartree-Fock method (RT-TDHF) therefore utilizes the STO-3G minimal basis, truncates long-range interactions and contains the approximations inherent to the Hartree-Fock method. These limitations are to a large part of a systematic nature - especially for very similar structures as the considered DNA samples - and can be compensated by considering the large data set of DNA spectra. Regarding the systematic correction of errors in TDHF and TDDFT calculations, we refer the interested reader to references [14, 15] and for the concrete parametrization and validation of our framework to reference [16].

For the computation of DNA absorption spectra specifically, we use a sequence containing the four base pairs adenine (A), cytosine (C), guanine (G), and thymine (T) as the starting point. Atomic coordinates are then generated for this sequence using the nucleic acid building tool of UCSF ChimeraX [17–19]. Hydrogen atoms are added to the structure which is then used as the starting point for the absorption spectrum calculation. Due to the negative charges near the phosphate in the DNA backbone, additional electrons have to be added. We add 2n-2 electrons for a DNA sample with n base pairs and therefore n-1 backbone connections on each chain.

A real-time approach is used by us as it scales more favorable than linear-response formalisms which can scale up to $𝒪\left(n^{6}\right)$ if all roots are considered, whereas real-time approaches have matrix operations at an $𝒪\left(n^{3}\right)$-cost as the bottleneck. The theoretical $𝒪\left(n^{4}\right)$-scaling of the Hartree-Fock method, caused by the electron repulsion integrals, can be reduced to $𝒪\left(n^{2}\right)$ or 𝒪(n) [20].

In the RT-TDHF calculations, the system is excited with a small initial electric field pulse and then propagated over 500 atomic units of time (12.1 fs). The propagation is done with a predictor-corrector scheme [21] and runs over 2000 steps. The dipole moments d are calculated at each step and saved. To compute the absorption, a Fourier transform of the dipole moments is taken. Then, the absorption corresponds to the imaginary part of this Fourier transform divided by a Fourier transform of the electric field pulse, multiplied with the frequency ω and summed over all three spatial dimensions:

(1) $$A\left(\omega \right)=\sum_{\alpha =x,y,z}-\frac{4\pi \omega }{3c}I\left(\frac{\int_{0}^{t_{\text{max}}}e^{i\omega t}d_{\alpha }\left(t\right)e^{-\gamma t^{2}}\;dt}{\int_{0}^{t_{\text{max}}}e^{i\omega t}E_{\alpha }\left(t\right)\;dt}\right),$$

with an attenuation factor $\gamma =1.5\cdot 10^{-4}$ a.u. and the speed of light c, the integral is then numerically evaluated with the data obtained for the time steps. Note that an attenuation factor in the form of an exponential function is necessary since we only have a finite signal in the time domain. Nonphysical oscillations are therefore suppressed, often a decreasing exponential is used. Here, we use a Gaussian function since the resulting signal in the frequency domain then decreases faster which suits the behaviour of the DNA spectra in the UVB region better.

During the time propagation, the main part of the computational cost is caused by the matrix operations, especially the matrix exponentiation, which are computed every time step. Additionally, the computation of the electrostatic potential has to be performed at each step as well since the electron density changes due to the quantum-mechanical time evolution. Here, we use the Hartree-Fock method as it allows a very fast calculation of the electrostatic potential which otherwise would become costly to evaluate due to the large three-dimensional space that the DNA samples take. In the Hartree-Fock formalism we can store the relevant integrals for the computation of the Fock matrix as their number is between 100 million and a few billion which is within the scope of available RAM for most modern CPU-based compute nodes. Furthermore, cutting long-range interactions increases the speed of this part of the calculation even more while often not significantly influencing the results in RT-TDHF calculations [16]. On the other hand, an inclusion of all interactions is still possible and would increase the computation time of the electrostatic potentials by a few times, depending on the system size. Testing this for the DNA dimers showed only small deviations from the results with cut-offs, well within the errors that are to be expected by the limitations of minimal-basis Hartree-Fock regardless. In summary, the RT-TDHF approach ensures a much faster construction of the Hamiltonian matrix which has been identified as the bottleneck of RT-TDDFT calculations for large systems [21].

Overcoming above-mentioned limitations can be done by performing systematic corrections which work especially well if a class of similar molecules is considered. For this correction, a linear function is calculated as a regression based on two sets of experimental and theoretical peak positions and then applied to the peak wavelengths [14, 15]. In this work, we apply the linear function over an interval of wavelengths since we are more interested in the shape of the spectrum and do not have a peak in the relevant UVB region to use for correction purposes. To do so, we use the experimental spectrum of DNA between 220 and 320 nm as a reference [22], with a special focus on modeling the UVB absorption (at around 300 nm), since we want to compare the DNA samples at this wavelength later. The theoretical spectrum used for this comparison is computed as the average spectrum of all 256 DNA tetramers.

Key features of the absorption spectrum of DNA are a minimum at 230 nm, a maximum at 260 nm and a decrease to nearly zero absorption at 320 nm. Additionally, the ratio of the absorption at 260 nm and at 280 nm should be near 1.8 for pure DNA samples and the ratio between 260 nm and 230 nm is expected to be around 2.0. Finally, since we are interested in the UVB absorption, we furthermore want to have a ratio between the peak and the absorption at 300 nm of approximately 0.05.

By testing different scaling factors and offsets, we observe that all of these conditions get closely satisfied with the correction function $\lambda_{\text{corr}}=1.1\cdot \lambda_{\text{calc}}+20\;\text{nm}$. The resulting spectrum of all 256 samples and their average and comparison to experimental data [22] is displayed in Figure 2 with an enlarged display of the relevant UVB region.

In addition to the systematic investigation of the UV absorption of smaller DNA structures, it is also interesting to consider larger DNA samples. We therefore computed the absorption of ten randomized DNA structures with eleven base pairs since at this amount of base pairs nullomers start to occur in the human genome.

During the generation of the randomized samples the only constraint was that on average the content of the different base pairs corresponds to the distribution in the human genome (around 30% adenine, 30% thymine, 20% cytosine and 20% guanine).

For the nullomers, the used sequences are CGCTCGACGTA, GTCCGAGCGTA, CGACGAACGGT, CCGATACGTCG, TACGCGCGACA, CGCGACGCATA, TCGGTACGCTA, TCGCGACCGTA, CGATCGTGCGA, and CGCGTATCGGT. These are taken from Table 3 of Ref. [5]. Note that the sequence CG and in general that cytosine (C) and guanine (G) occur very often, in contrast to the average in the human genome which only contains C and G to in total 40.89% [23].

To generate the corresponding synonyms, we used the nullomer sequences and changed them to sequences with the same meaning. During the translation of DNA, three base pairs always correspond to a certain amino acid. As there are more DNA trimers (64) than amino acids (20), a sequence of amino acids often can be expressed by different combinations of DNA trimers (so-called codons). We translated the used nullomers to DNA sequences by cutting the sequence in four codons. Each codon was translated to its corresponding amino acid and the amino acid was translated back to the codon which is used most commonly for this specific amino acid in the human genome. Note that there are three different ways of how a DNA sequence can be split into codons, depending on the starting point of reading - this is called the reading frame. Here, our reading frame starts with the first base pair. The fourth codon then misses one base pair which was replaced by the most likely codon given the first two base pairs.

Investigating the 30 samples of longer DNA strands (random, nullomers and synonyms) reveals systematic differences in the UV absorption between these three classes (see Figure 2). The random DNA samples show the largest deviations from each other regarding their absorption around 300 nm, as indicated by the orange area, which denotes one standard deviation. For the nullomers and their synonyms, the absorption curves are less spaced out, with the average nullomer absorption beyond the standard deviation of the random DNA absorption spectra. It is highly likely that the increased absorption in nullomers is caused by the high content of cytosine and guanine in these structures.

The average of the UV absorption of the synonym sequences lies significantly below the average of the nullomer absorption, but is still located among the random DNA samples with higher absorption. This indicates that while more commonly used codons have a lower susceptibility to UV radiation, the higher amount of absorption in nullomers cannot be compensated completely by using a different expression for the genes. This can be considered sensible since there is only some flexibility during the translation of the nullomer sequences to synonyms. The different codons for a single amino acid often only differ in one base pair which leads to parts of the sequence remaining the same.

Next, we compare the absorption of all possible DNA monomers, dimers, trimers and tetramers (see Figure 3). Additionally, it is considered how often these sequences occur in the human genome. For all four classes, an inverse correlation between their occurrence and their absorption at 300 nm is found. The correlation coefficients between these two variables are −0.937, −0.633, −0.705, and −0.692 for one, two, three, and four base pairs, respectively, indicating a negative correlation. Conversely, positive correlation coefficients are observed between the occurrence count and the inverse of the absorption, with values of 0.857, 0.707, 0.745, and 0.721.

In the scatter plot in Figure 3, an island can be found near the origin. The base pair combinations in this region all contain the sequence CG which also occurs very often in the nullomer sequences. This particular dimer occurs only rarely in genomes as it is associated with a higher mutation rate: The methylated cytosine in these sites can get deaminated into thymine, leading to a base pair mismatch [24]. The low occurrence of the sequence CG can also be seen as the red bars in Figure 3, right.

Finally, we consider where the absorption of UV light happens in the DNA sample. This can give information of which parts of the DNA (backbone or base pairs) are involved in the absorption processes and for longer sequences it also shows which nucleotides absorb the most.

To do so, we also calculate a Fourier transform of the dipole moments of the individual basis functions to obtain their respective contributions to the overall absorption. These are linked with the atoms of the basis functions and their constant offset which is still present at zero absorption is subtracted. This generates information about the participation of individual atoms at the absorption at a certain wavelengths.

In Figure 4, the above-mentioned method is used to visualize the absorption at 300 nm in the DNA dimer TG which highlights that in this case guanine and cytosine absorb more light than thymine and adenine. The absorption of the first nullomer sample is also visualized in Figure 4. In the top-down view, we observe that absorption happens mostly in the base pairs rather than the DNA backbone. While almost all CG base pairs absorb light, three adenine nucleotides are also involved in the absorption. Hence, there is not a general rule for the absorption of larger DNA samples as their absorption appears more complex than the sum of their nucleotides.

Over all samples considered, we do however observe the trend that cytosine- and guanine-rich DNA has increased absorption in the UVB region at 300 nm while adenine- and thymine-rich DNA in general exhibits lower absorption.

The human genome exhibiting a lower content of C and G than A and T is a likely explanation for the correlation between sequence occurrence and respective UV absorption discussed in Figure 3.

There are multiple possible explanations for the cause of low GC content in the human genome [24–26]. These include mutation biases towards A and T [27] and deamination of cytosine [28], however, the phenomenon of low GC content is not fully understood. Potentially, the higher absorption of cytosine and guanine could be another reason for low GC content being evolutionary advantageous as it reduces the susceptibility of the genome to damage and mutations caused by UV radiation. Based on experimental results it has been postulated by Matallana-Surget et al. that CG-rich genomes lead to an increased occurrence of UV-radiation induced mutations [29] and Sutherland et al. found a correlation between the GC content of genomes of different species and corresponding DNA absorption [30]. These results match well with the theoretical investigation of individual DNA sequences in this study. Similarly, the reason for nullomers in the human genome is not solved as well with natural selection and the high mutation rate of the sequence CG discussed as potential causes [6]. Here, the high UVB absorption of these sequences could be an additional reason for avoiding them in the human genome.

In summary, we performed a systematic theoretical investigation of UV absorption in different DNA configurations showing that AT-rich DNA is favorable for avoiding UV radiation damage. Future research could include absorption spectra calculations of RNA or interactions of DNA and small molecules. Additionally, molecular dynamics simulations with excited states, investigations of the changes in the electron density during the absorption and searches for DNA sequences with very high or very low UV absorption could be possible. We hereby hope to provide a starting point for investigating electronic processes in larger DNA samples with fully quantum-mechanical methods.

## Figures and Tables

**FIG. 1.**
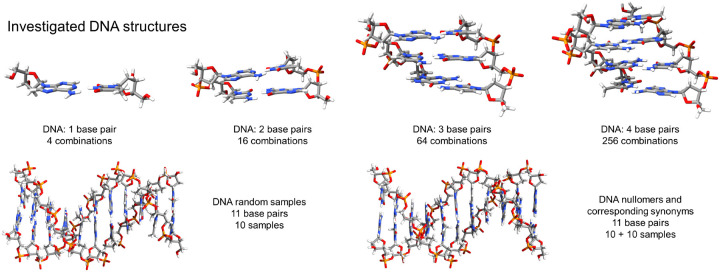
Atomistic visualization of the studied DNA samples. Top row: Examples for DNA monomers, dimers, trimers and tetramers. Bottom row: 11-base pair DNA samples used for computing the UV absorption spectra of nullomers, synonyms and random DNA. One nullomer and one random sample are depicted, only subtle differences can be seen due to the different base pair sequences. Visualization with UCSF ChimeraX.

**FIG. 2.**
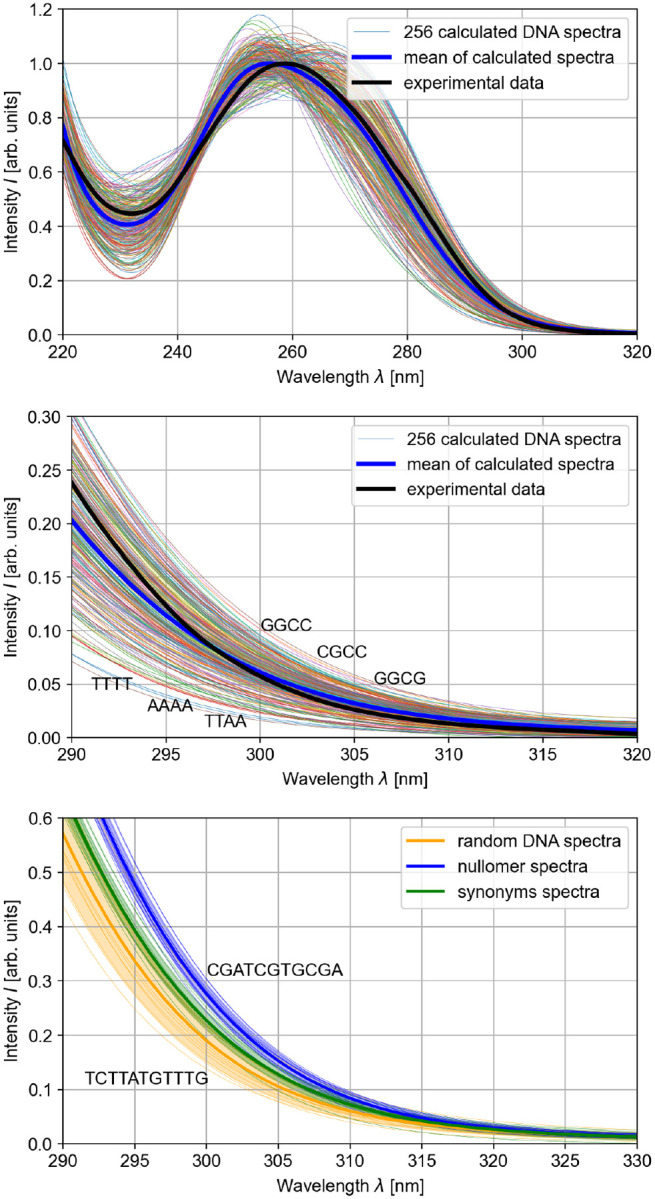
Top: The corrected spectra of the 256 possible DNA tetramers are shown as colored lines. Additionally, the mean of these spectra is depicted (blue) and compared to an experimental spectrum of DNA (black) [22]. Middle: The results from the upper panel are shown in the region between 290 and 320 nm with the sequences with the lowest and highest absorption highlighted. Bottom: The absorption spectra of the ten investigated nullomers (blue), their synonyms (green) and of ten random DNA samples (yellow) are shown. The individual spectra are shown as narrow lines, the averages as wide lines and the standard deviations as colored background.

**FIG. 3.**
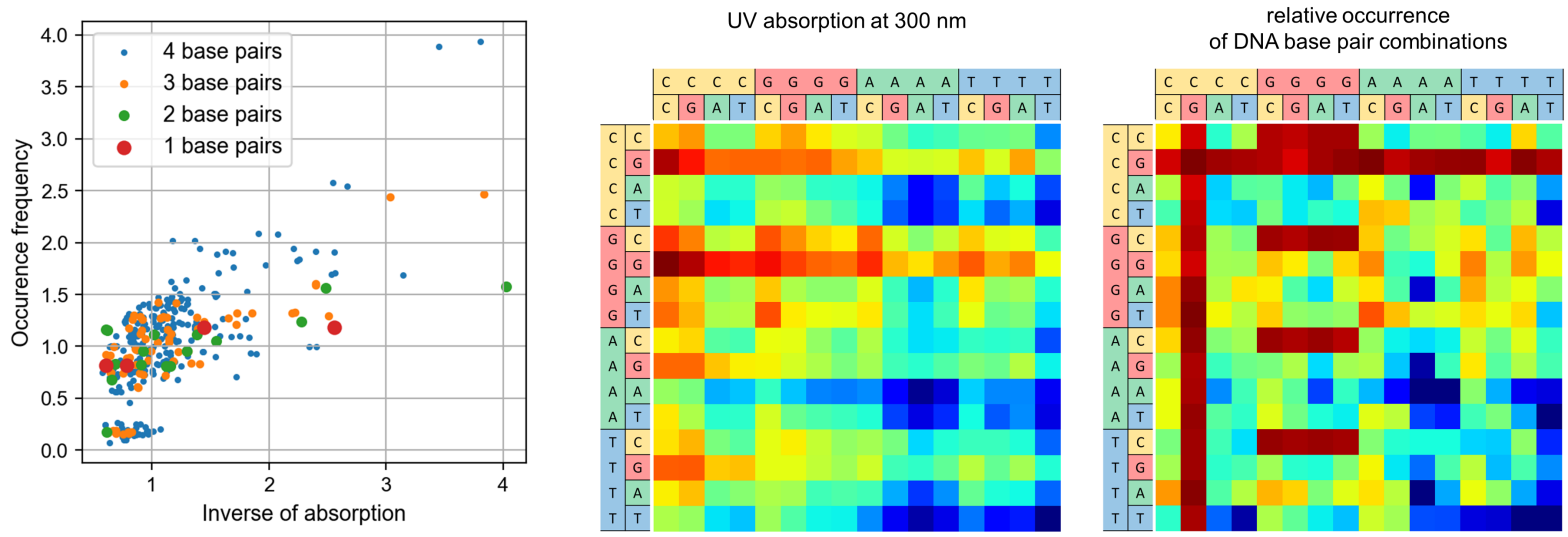
Left: The normalized occurrence frequency of DNA monomers (red), dimers (green), trimers (yellow) and tetramers (blue) are plotted against the normalized inverse of their absorption at 300 nm. Middle/right: UV absorption at 300nm and relative occurrence of all 256 DNA tetramers, each visualized as a single square. Blue corresponds to low absorption/high occurrence and red to high absorption/low occurrence. Occurrence is truncated at 2 · 10^7^ for visualization purposes due to exceptional high occurrence of some A/T-rich sequences.

**FIG. 4.**
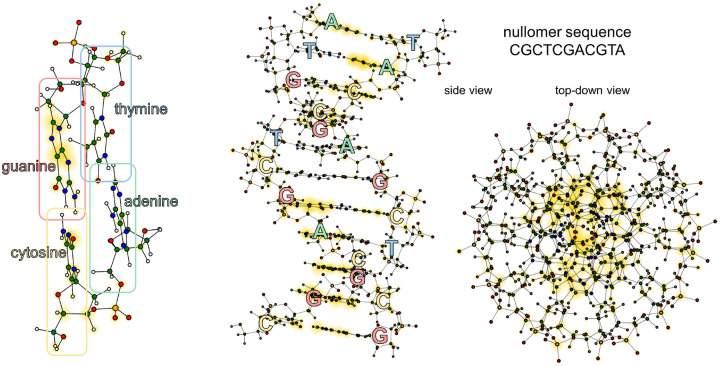
Relevant absorption regions of a DNA dimer (TG, left) and the first nullomer sample (CGCTCGACGTA, middle and right, in different perspectives). Atoms participating in the absorption at 300 nm are highlighted with yellow circles with the size of the circles proportional to their contribution.
